# Corneal Astigmatism Measurements Comparison among Ray-Tracing Aberrometry, Partial Coherence Interferometry, and Scheimpflug Imaging System

**DOI:** 10.1155/2020/3012748

**Published:** 2020-04-01

**Authors:** Yaqin Zhang, Jing Dong, Suhua Zhang, Bin Sun, Xiaoliang Wang, Maolong Tang, Xiaogang Wang

**Affiliations:** ^1^Shanxi Eye Hospital, Taiyuan, Shanxi, China; ^2^The First Hospital of Shanxi Medical University, Taiyuan, Shanxi, China; ^3^School of Aeronautics and Astronautics, Shanghai Jiao Tong University, Shanghai, China; ^4^Casey Eye Institute, Oregon Health and Science University, Portland, OR, USA

## Abstract

**Purpose:**

To investigate interdevice agreement among corneal topography/ray-tracing aberrometry (iTrace), partial coherence interferometry (IOLMaster), and Scheimpflug imaging (Pentacam) for the measurement of corneal astigmatism.

**Methods:**

The analysis included 90 eyes of 90 subjects without ocular disease. The main outcome measures were corneal cylinder power and axis of astigmatism. All corneal astigmatism measurements were converted to two perpendicular components by using vector analysis. Interdevice agreement was assessed using Bland–Altman analysis, paired sample *t*-test, and one-way analysis of variance.

**Results:**

No significant interdevice difference existed in the astigmatism magnitude, cardinal component, and oblique component (all *P* > 0.05). On comparing iTrace wavefront and simulated keratometry (SimK) astigmatism, significant differences were observed in the astigmatism magnitude and oblique component (both *P* < 0.01), but not in the cardinal component (*P*=0.687). On comparing Pentacam pupil 3 mm and corneal vertex 3 mm axial astigmatism, significant difference was observed in the astigmatism magnitude (*P* < 0.001), but not in the cardinal and oblique components (both *P* > 0.05).

**Conclusions:**

The iTrace, IOLMaster, and Pentacam devices could be used interchangeably for corneal astigmatism measurement. However, the measurement difference in iTrace wavefront and SimK astigmatism and Pentacam pupil 3 mm and vertex 3 mm axial astigmatism should be considered in clinic practice.

## 1. Introduction

Preoperative corneal astigmatism greater than 0.5 diopters (D) should be considered for correction to gain clearer vision in case of cataract surgery [1]. Precise assessment of preoperative corneal astigmatism plays an important role in choosing the intraocular lens (IOL) type (toric IOL or not), clear corneal incision location (at the steepest axis or not), and peripheral corneal relaxing incisions for patients with cataract. Moreover, effective correction of preexisting corneal astigmatism depends greatly on its accurate preoperative measurement.

As a standard device for corneal astigmatism measurement, the keratometer is widely used in ophthalmic clinics. Different types of keratometers may offer different astigmatism values, which might provide different toricity choices for toric IOLs [2–6]. The potential measurement difference and different IOL toricity calculation formulas may cause under- or overcorrection of corneal astigmatism postoperatively [7].

IOLMaster 500 (Carl Zeiss Meditec, Germany), based on partial coherence interferometry, measures anterior corneal astigmatism and curvature by analyzing the real position of each pair of reflection spots (six spots of light arranged in a hexagonal pattern) from the anterior surface of the cornea with a diameter ring of around 2.3 to 2.5 mm. Some authors even considered it the gold standard for assessing new topographers or keratometers [8, 9]. Previous studies have also demonstrated that the astigmatism data obtained using IOLMaster provide good outcomes when used for toric IOL selection [3, 10].

The Pentacam Scheimpflug imaging system (OCULUS, Wetzlar, Germany) can capture 25 to 50 images by rotating 360° in one examination. It also can image and perform automated measurement of the anterior and posterior corneal surfaces. Anterior corneal astigmatism data, as a computerized value focused on the anterior 3.0 mm of the cornea, can be centered on the corneal vertex or pupil [11]. A few studies have compared keratometry and astigmatism values (centered on the corneal vertex) measured using IOLMaster versus Pentacam, but have reported controversial results, especially about corneal astigmatism measurement [2, 12–14].

iTrace ray-tracing aberrometry (Tracey™ Technologies, Texas, USA), based on corneal topography, can provide simulated keratometry (SimK) and astigmatism data with a diameter of 3.0 mm centered on the corneal vertex [15]. Moreover, it can provide the wavefront astigmatism value of the cornea, which is recommended for toric IOL calculation via the HOYA iTrace Surgical Workstation, by projecting a bundle of rays parallel to the device axis into the eye by using a laser ray-tracing method [16].

Comparing the corneal astigmatism values obtained using different devices on the same eye can provide clues to understanding the trends of current corneal power measurement systems. Therefore, the primary purpose of this study was to investigate the agreement among IOLMaster, Pentacam, and iTrace with respect to corneal astigmatism measurements. The secondary aim was to evaluate (1) the interchangeability of corneal astigmatism values centered on the corneal vertex and pupil by using Pentacam and (2) the interchangeability of corneal astigmatism values between iTrace simulated values and corresponding wavefront values by using iTrace.

## 2. Methods

### 2.1. Subjects

This study was performed at the Shanxi Eye Hospital (Taiyuan, Shanxi, China). The research protocol was approved by the institutional review board of Shanxi Eye Hospital and carried out according to the tenets of the Declaration of Helsinki. Written informed consent was obtained from each subject after explaining the nature of this study.

Consecutive patients diagnosed with cataract were retrospectively enrolled between April 2017 and July 2017. The inclusion criteria were as follows: senile cataracts, no systemic disease, nuclear cataract grade 3 (Lens Opacities Classification System III), no pathological alteration of the anterior segment (such as keratoconus, zonular dialysis, pseudoexfoliation syndrome, or corneal opacity), no retinal diseases impairing visual function, and no previous anterior or posterior segment surgery.

### 2.2. Data Acquisition

Corneal astigmatism was measured using the same sequence of IOLMaster, iTrace, and Pentacam for each eye. In accordance with the user guidelines of each device, effective measurements (measurement quality check list was ok for Pentacam; no rejection point and Placido rings were unbroken for iTrace; three K readings difference would be less than a quarter-diopter for IOLMaster) were used in the final analysis. The software used was version 1.20r36 for Pentacam, version 6.1.0 for iTrace, and version 7.5 for IOLMaster 500. All measurements were performed in a semidark room. The subjects were asked to place their chin on the chin rest and press the forehead against the forehead strap. The eye was then aligned to the corneal topographic axis by using a central fixation light or target. The subjects were instructed to perform a complete blink before each measurement. A single trained operator performed all of the examinations using the three devices.

### 2.3. Vector Analysis of Astigmatism

Vector analysis was used to compare the corneal astigmatism values from the three devices [17]. The astigmatism values were decomposed into two perpendicular components as follows:

(1a) $$\begin{array}{c} X=A\;\cos\left(2\alpha \right), \end{array}$$

(1b) $$\begin{array}{c} Y=A\;\sin\left(2\alpha \right), \end{array}$$

where *X* is the cardinal component, *Y* is the oblique component, *A* is the astigmatism magnitude in diopters, and *α* is the astigmatism axis in degrees.

### 2.4. Statistical Analysis

Statistical analyses were performed using commercial software (SPSS for Windows, Version 13.0; SPSS Inc., Illinois, USA). The Kolmogorov–Smirnov test was used to assess data normality. Based on the data normality test result, one-way analysis of variance (ANOVA) or nonparametric tests were utilized to compare the corneal astigmatism values among the three devices. The statistical significance of the intradevice difference was investigated using the paired two-tailed *t*-test. The interdevice agreement was evaluated using Bland–Altman analysis, and the interdevice differences were plotted against their means with 95% limits of agreement (LoAs). All tests had a significance level of 5%.

## 3. Results

Ninety eyes of 90 subjects were randomly included in the final study. Demographics of the study population are summarized in Table 1.

The mean simulated astigmatism and corneal power measurements obtained using IOLMaster, iTrace, and Pentacam are listed in Table 2.

**Figure 1:**
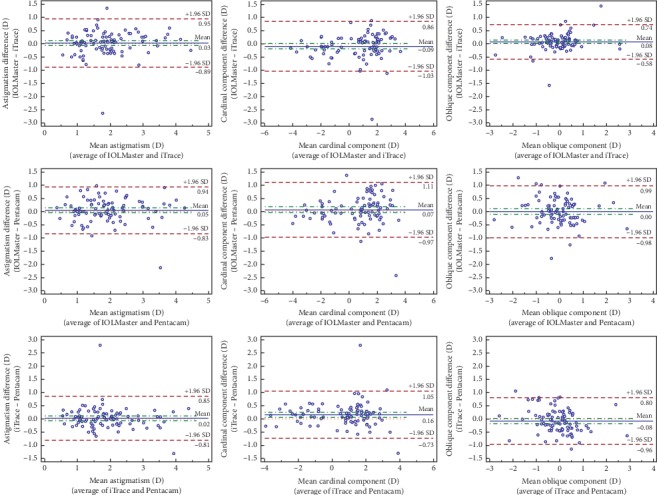
Bland–Altman analysis of agreement among IOLMaster, iTrace, and Pentacam for the astigmatism magnitude (panels A, D, and G), cardinal component (panels B, E, and H), and oblique component (panels C, F, and I). The mean difference is demonstrated by the horizontal blue solid line, and the 95% limit of agreement is shown by the brown dotted lines. D = diopters; SD = standard deviation.

The Kolmogorov–Smirnov test result demonstrated that the flat keratometry of the three devices and the steep keratometry and mean keratometry of IOLMaster and iTrace passed the test of normality (all *P* > 0.05). However, the other values failed the test of normality (all *P* < 0.05). The Kruskal–Wallis nonparametric test revealed no significant difference among the three devices for the astigmatism magnitude (*P*=0.901), cardinal component (*P*=0.664), oblique component (*P*=0.635), K flat (*P*=0.310), K steep (*P*=0.335), and K mean (*P*=0.294). For the corneal astigmatism magnitude, cardinal component, and oblique component, the 95% LoAs between every two devices were within the clinically relevant margins of discrepancy (Figure 1 and Table 3).

For iTrace astigmatism comparison, the wavefront astigmatism magnitude was about 0.10 D higher than that of the simulated values (Table 4) (*P* < 0.001). In contrast, the wavefront astigmatism oblique component was around 0.04 D lower than that of the simulated values (Table 4) (*P*=0.001).

For Pentacam simulated astigmatism comparison centered on the corneal vertex and pupil, the astigmatism magnitude centered on the pupil was about 0.16 D lower than that of the corneal vertex (Table 5) (*P* < 0.001). No significant differences were found for vector terms between the values centered on the pupil and corneal vertex (Table 5) (*P* > 0.05).

## 4. Discussion

Proper patient selection and precise measurement of corneal astigmatism are two key factors in toric IOL cataract surgery. All toric IOL calculators require accurate preoperative measurements of corneal astigmatism to decide the final toric IOL power and the meridian of IOL alignment. Different keratometry and corneal topography devices are important for obtaining precise corneal astigmatism vector values. Therefore, the purpose of this study was to evaluate the comparability and interchangeability of astigmatism measurements obtained using the three commonly used clinical devices.

Bland–Altman analysis revealed good agreement among IOLMaster, iTrace, and Pentacam. By using subjective manifest refraction as a standard, iTrace demonstrated reliable and reproducible refractive error measurement [18]. Thus, iTrace was used as a standard in this study. Good agreement was observed between Pentacam and iTrace, as demonstrated by the small 95% LoA spread. Good agreement was also observed between IOLMaster and Pentacam, as well as IOLMaster and iTrace. In a previous study comparing the corneal astigmatism measurements obtained using different devices (including IOLMaster, manual keratometry, Atlas corneal topography, and Galilei dual Scheimpflug analyzer), IOLMaster showed the highest corneal astigmatism values; this tendency was consistent with our current findings [19]. This may be because IOLMaster measures a relatively smaller corneal diameter than does Pentacam or iTrace [2, 20]. The mean difference of corneal astigmatism magnitude among the three devices ranged from 0.02 D to 0.05 D, which was much lower than the toric IOL 0.5-D gradation of cylinder power at the corneal plane. Therefore, the corneal astigmatism measurement agreement among the three devices was deemed good overall.

The iTrace ray-tracing wavefront mean corneal astigmatism values were about 0.1 D higher than those of the iTrace SimK values, which was consistent with the findings of a previous study [16]. The reasons for the measurement inconsistency mainly include the following: (1) different measurement area, ray-tracing wavefront corneal astigmatism measurement focuses on the entire area of the cornea, which is much bigger than the 3 mm measurement area of SimK; and (2) different measurement technology, the former uses ray-tracing technology, which measures by projecting a near-beam into the eye, whereas the latter measurement is based on the analysis of a Placido ring [16].

Owing to the easy detection of pupil boundaries by eye-tracking devices, the pupil center may be the most commonly used centration method. However, the pupil center is unstable and shifts around 0.2 to 0.5 mm with different pupil sizes; hence, a more stable morphologic reference is advisable [21–23]. The corneal vertex, mostly located nasally relative to the pupil center, is the highest point of the cornea during gaze at the fixation target. It is also known as the first Purkinje reflex or vertex normal. It is the point where the corneal topographic axis hits the cornea [11]. Our finding also demonstrated higher toricity values (astigmatism magnitude: 0.16 D) when centered on the corneal vertex than when centered on the pupil center. Using the criterion of 0.5 D, no detectable difference was observed between the measurements obtained using the pupil center or corneal vertex as the reference center.

A limitation of this study was that we included no subgroups showing with-the-rule or against-the-rule corneal astigmatism. If the eyes are grouped into different subgroups, the results may differ.

In summary, the present study evaluated the comparability of anterior corneal astigmatism measurements from three devices. Bland–Altman analysis demonstrated good agreement among these three devices. Moreover, the measurement difference between iTrace wavefront astigmatism and SimK astigmatism, and that between Pentacam pupil 3 mm and corneal vertex 3 mm axial astigmatism, may not be clinically meaningful.

## Figures and Tables

**Table 1:** Patient's demographics.

Characteristics	No.
Eyes (% right eyes)	48 (53.3%)
Age, years (mean ± SD)	69.0 ± 10.1
Sex (% male)	43 (47.8%)
Axial length, mm (mean ± SD)	23.51 ± 1.25
Anterior chamber depth, mm (mean ± SD)	3.07 ± 0.40

SD = standard deviation.

**Table 2:** Mean simulated corneal astigmatism and corneal power measurements obtained using IOLMaster, Pentacam, and iTrace.

	IOLMaster at 2.3 mm	iTrace at 3 mm	Pentacam at 3 mm (corneal vertex)
Astigmatism magnitude (D)	1.93 ± 0.88	1.89 ± 0.84	1.88 ± 0.90
Astigmatism cardinal	0.65 ± 1.84	0.74 ± 1.78	0.60 ± 1.79
Astigmatism oblique	−0.03 ± 0.86	−0.11 ± 0.78	−0.05 ± 0.89
K flat	44.12 ± 1.69	43.75 ± 1.71	44.06 ± 1.67
K steep	46.05 ± 1.73	45.65 ± 1.72	45.93 ± 1.74
K mean	45.09 ± 1.65	44.71 ± 1.66	45.00 ± 1.65

D = diopters; K = keratometry.

**Table 3:** Comparison of corneal astigmatism values between devices.

Device	Correlation between devices (*P*)			Mean difference (D) ± SD			95% CI			95% of LoA spread (D)		
	Magnitude	Cardinal component	Oblique component	Magnitude	Cardinal component	Oblique component	Magnitude	Cardinal component	Oblique component	Magnitude	Cardinal component	Oblique component
IOLMaster vs. iTrace	0.853 (<0.0001)	0.965 (<0.0001)	0.920 (<0.0001)	0.03 ± 0.47	−0.09 ± 0.48	0.08 ± 0.34	−0.07, 0.13	−0.19, 0.01	0.01, 0.15	1.84	1.89	1.32
IOLMaster vs. Pentacam	0.870 (<0.0001)	0.957 (<0.0001)	0.839 (<0.0001)	0.05 ± 0.45	0.07 ± 0.53	0.00 ± 0.50	−0.04, 0.15	−0.04, 0.18	−0.10, 0.11	1.77	2.08	1.97
iTrace vs. Pentacam	0.881 (<0.0001)	0.967 (<0.0001)	0.868 (<0.0001)	0.02 ± 0.42	0.16 ± 0.46	−0.08 ± 0.45	−0.07, 0.11	0.07, 0.26	−0.17, 0.01	1.66	1.78	1.76

CI = confidence interval; D = diopters; LoA = limit of agreement; SD = standard deviation.

**Table 4:** Difference between the wavefront corneal astigmatism and simulated corneal astigmatism measurements obtained using iTrace.

	Wavefront astigmatism	Simulated astigmatism	Difference (W − S) ± SE	*P* ^*∗*^
Astigmatism magnitude (D)^#^	2.00 ± 0.87	1.89 ± 0.84	0.10 ± 0.02	<0.001
Astigmatism cardinal	0.75 ± 1.88	0.74 ± 1.78	0.01 ± 0.02	0.687
Astigmatism oblique^#^	−0.15 ± 0.83	−0.11 ± 0.78	−0.04 ± 0.01	0.001

D = diopters; S = simulated astigmatism; SE = standard error; W = wavefront astigmatism. ^*∗*^Paired two-tailed *t*-test. ^#^Statistically significant at the 5% level.

**Table 5:** Difference between 3 mm axial corneal astigmatism measurements centered on the pupil and corneal vertex obtained using Pentacam.

	Astigmatism centered on the pupil	Astigmatism centered on the vertex	Difference (*P* − V) ± SE	*P* ^*∗*^
Astigmatism magnitude (D)^#^	1.72 ± 0.83	1.88 ± 0.90	−0.16 ± 0.03	<0.001
Astigmatism cardinal	0.58 ± 1.62	0.60 ± 1.79	−0.02 ± 0.03	0.612
Astigmatism oblique	−0.04 ± 0.85	−0.05 ± 0.89	0.01 ± 0.01	0.480

V = astigmatism centered on the corneal vertex; D = diopters; *P* = astigmatism centered on the pupil; SE = standard error. ^*∗*^Paired two-tailed *t*-test. ^#^Statistcally significant at the 5% level.

## Data Availability

The data used to support the findings of this study are available from the corresponding author upon request.

## Additional Points

What Was Known. (i) IOLMaster, iTrace, and Pentacam can be used interchangeably to determine corneal astigmatism before toric IOL implantation. (ii) Some devices have been used to measure corneal astigmatism in the calculation of toric IOL implantation. Analysis methods, including iTrace wavefront astigmatism and Pentacam simulated astigmatism centered on the pupil or corneal vertex, have not been evaluated simultaneously in the preoperative evaluation for toric IOL calculation. What This Paper Adds. (i) iTrace wavefront astigmatism is higher than iTrace simulated astigmatism. (ii) Pentacam simulated astigmatism centered on the corneal vertex is higher than the value centered on the pupil.
